# Wee1 inhibition potentiates Wip1-dependent p53-negative tumor cell death during chemotherapy

**DOI:** 10.1038/cddis.2016.96

**Published:** 2016-04-14

**Authors:** V Clausse, A R Goloudina, B Uyanik, E Y Kochetkova, S Richaud, O A Fedorova, A Hammann, M Bardou, N A Barlev, C Garrido, O N Demidov

**Affiliations:** 1 INSERM UMR 866, Laboratoire d'excellence ARC, Dijon, France; 2 University of Burgundy, Dijon, France; 3 Institute of Cytology, RAS, St. Petersburg, Russia; 4 Anticancer Center Georges François Leclerc, Dijon, France

## Abstract

Inactivation of p53 found in more than half of human cancers is often associated with increased tumor resistance to anti-cancer therapy. We have previously shown that overexpression of the phosphatase Wip1 in p53-negative tumors sensitizes them to chemotherapeutic agents, while protecting normal tissues from the side effects of anti-cancer treatment. In this study, we decided to search for kinases that prevent Wip1-mediated sensitization of cancer cells, thereby interfering with efficacy of genotoxic anti-cancer drugs. To this end, we performed a flow cytometry-based screening in order to identify kinases that regulated the levels of *γ*H2AX, which were used as readout. Another criterion of the screen was increased sensitivity of p53-negative tumor cells to cisplatin (CDDP) in a Wip1-dependent manner. We have found that a treatment with a low dose (75 nM) of MK-1775, a recently described specific chemical inhibitor of Wee1, decreases CDDP-induced H2AX phosphorylation in p53-negative cells and enhances the Wip1-sensitization of p53-negative tumors. We were able to reduce CDDP effective concentration by 40% with a combination of Wip1 overexpression and Wee1 kinase inhibition. We have observed that Wee1 inhibition potentiates Wip1-dependent tumor sensitization effect by reducing levels of Hipk2 kinase, a negative regulator of Wip1 pathway. In addition, during CDDP treatment, the combination of Wee1 inhibition and Wip1 overexpression has a mild but significant protective effect in normal cells and tissues. Our results indicate that inhibition of the negative regulators of Wip1 pathway, Wee1 and Hipk2, in p53-negative tumors could potentiate efficiency of chemotherapeutic agents without concomitant increase of cytotoxicity in normal tissues. The development and clinical use of Wee1 and Hipk1 kinase chemical inhibitors might be a promising strategy to improve anti-cancer therapy.

Various approaches in modern day anti-cancer treatment rely on DNA damage response (DDR) system so as to target rapidly dividing cancer cells by direct or indirect DNA damage.^1^ In the core of the DDR system is the tumor suppressor p53, a transcription factor which by modulating gene expression controls cell response to numerous stressors and determines cell fate.^2^ To overcome the negative supervision of oncogenic behavior by p53, tumor cells should neutralize p53 pathway to allow their uncontrolled growth.^3, 4^ The loss of p53 tumor-suppressor functions is occurring in more than half of human cancers and affects many pathways, such as growth, genomic stability, cell death and sensitivity to anti-cancer treatment.^5^ Indeed, p53-negative tumors are often resistant to anti-cancer treatment. Finding new strategies that could restore p53-negative cancer cell sensitivity to chemotherapeutic agents will help to improve the efficacy of anti-cancer therapy.

We have developed an approach based on the synthetic sensitivity phenomenon of p53-negative cancer cells in the presence of overexpressed Wip1 phosphatase.^6^ Importantly, this approach allows specific targeting of only cancer cells, but not normal tissues. This therapeutic approach of synthetic lethality has been described for the first time in the 1920s by Calvin Bridges in *Drosophila melanogaster*.^7^ When two genes are genetically linked but are involved in different pathways, inactivation of both of them results in synthetic lethality. Yet, individual ablation of either of them does not cause lethality.^8^ In general, synthetic lethality can also be caused by specific drugs that inactivate one of the genes, given that the other is already inactivated.^9^ Importantly, we have shown that synthetic lethality can be caused not only by inactivation of the genes, but also by overexpression of an epistatic gene in the absence of another gene. In support of this hypothesis, we have shown that in p53-negative tumor cells, overexpression of this gene can restore cell death signaling in cisplatin (CDDP)-treated p53-negative cells.^10^

In line with this, in p53-negative tumors, Wip1 overexpression acts differently than in tumors harboring wild-type p53. Wip1 overexpression could negatively affect tumor characteristics in p53-positive cancers, such as breast cancer, neuroblastoma, medulloblastoma, ovarian clear cell tumor, gastric carcinoma, pancreatic adenocarcinoma and chronic lymphocytic leukemia.^11, 12^ Moreover, Wip1-deficient mice have shown a tumor-resistant phenotype strictly dependent on the presence of wild-type p53.^13, 14^ Wip1 overexpression induces opposite effects depending on the p53 status of the cell. By regulating the Bax/Bcl-xL ratio in the absence of p53, Wip1 overexpression sensitizes p53-negative tumors to chemotherapy and induces a caspase-3-dependent apoptosis.^10^

On the contrary, in normal cells that harbor wild-type p53 tumor suppressor, Wip1 overexpression lowers the amplitude of signaling from damaged DNA to p53.^15^ Thus, by attenuating p53 acute response to DNA damage, Wip1 protects normal cells from cisplatin-induced apoptosis.

Two different vectors of Wip1 phosphatase activity in p53-negative and p53-positive backgrounds not only restore chemotherapy efficacy in the former, but also reduce cytotoxicity and side effects in normal tissues, widening the therapeutic window.

The phosphorylation status of histone H2AX is a critical marker of DDR. When phosphorylated on Ser139 (*γ*H2AX), it initiates DDR by recruiting DDR factors to the DNA-damaged site. Previously, we and other groups have shown that Wip1 induction decreased the level of histone *γ*H2AX phosphorylation in the course of DNA damage in tumor cell lines.^16, 17, 18, 19, 20^ We hypothesized that we could achieve p53-negative cells sensitization with simultaneous normal cell protection by targeting kinases that affect the Wip1–*γ*H2AX pathway, directly or indirectly.

Here, we have reported an example of two such kinases identified by us in a human siRNA screen, Hipk2 and Wee1. Wee1 is a tyrosine kinase and a major regulator of the G2 checkpoint, which controls entry into mitosis after DDR.^21^ It has been published that Wee1 inhibition can sensitize cells to cisplatin treatment, by forcing them to enter mitosis with unrepaired DNA and inducing a mitotic catastrophe.^22^ We found that a low subtoxic concentration of a Wee1 inhibitor affects H2AX phosphorylation after CDDP-induced DNA damage and potentiates Wip1-dependent CDDP toxicity towards p53-negative cells, permitting a significant reduction of the efficient CDDP dose.

## Results

### Kinase library screening

**Figure 1:**
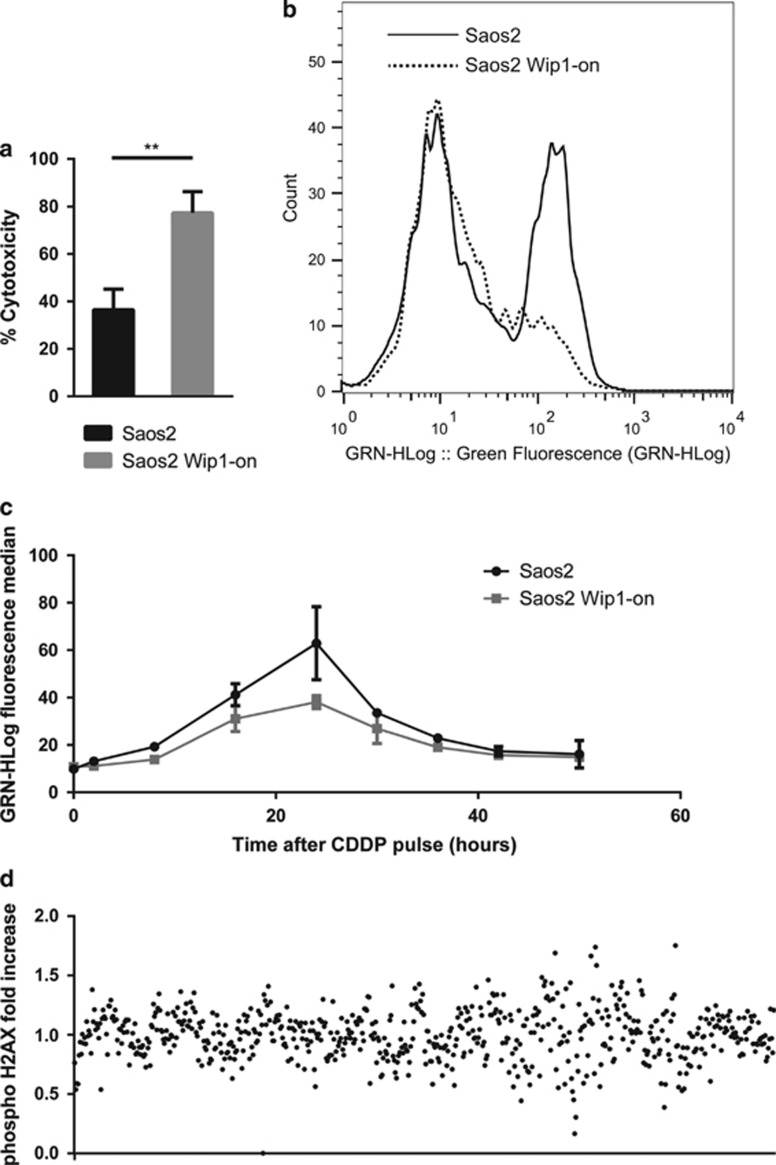
Wip1, a phosphatase that sensitizes p53-negative tumor cells to cisplatin is crucial for H2AX dephosphorylation after DNA damage, which can be used as a marker of Wip1 activity for a screening of human protein kinases interacting with DDR pathway. (**a**) Induction of Wip1 phosphatase in Saos2 osteosarcoma by a treatment of 1 *μ*g/ml doxycycline 24 h prior a 25 *μ*M cisplatin (CDDP) treatment for 72 h sensitizes these p53-negative cells to chemotherapy. (**b**) H2AX phosphorylation, the main marker of DNA damage, is decreased by Wip1 expression induced by a treatment of cells by 1 *μ*g/ml doxycycline 24 h prior a 25 *μ*M CDDP treatment for 28 h. (**c**) Kinetics of H2AX phosphorylation by flow cytometry with or without Wip1 induction by 1 *μ*g/ml doxycycline 24 h prior a 100 *μ*M CDDP pulse for 1 h. (**d**) High-throughput flow cytometry screening of H2AX phosphorylation following inhibition of the whole human kinome by siRNA reverse transfection in Saos2 osteosarcoma cells. Samples showing a modification of at least ±40% after a 25 *μ*M CDDP treatment for 28 h compared with controls transfected with non-targeting siRNA were arbitrarily selected as positive hits potentially acting in or through Wip1 pathway

Previously, it was reported that the increased *γ*H2AX levels correlates not only with more severe damage to DNA, but also with elevated DDR, including initiation of cell death program.^24^ Knowing the fact that, on one hand, Wip1 directly dephosphorylates *γ*H2AX and, on another hand, Wip1 increases cytotoxicity of cisplatin, we decided to verify that in p53-negative Saos2-Wip1-ON osteosarcoma cells, levels of p-Ser139 *γ*H2AX do not correlate with cisplatin-induced cell death. The induction of Wip1 in Saos2 cells increased significantly the cytotoxicity of a cisplatin treatment (*P*=0.0047), but levels of *γ*H2AX were lower than in control cells resistant to cisplatin without induction of Wip1 (Figure 1a, Supplementary Figure S1A).

During the process of DDR, H2AX is directly phosphorylated on Ser139 by several kinases such as ATM, ATR or DNA-PK.^25, 26, 27^ Several phosphatases including Wip1 were reported to be able to remove the phosphate group from this site, making this DNA damage marker turn back to the status OFF. Therefore, we analyzed several timepoints after administration of cisplatin in order to find the critical window in which the effect of Wip1 induction on *γ*H2AX dephosphorylation was at its maximum level. The most significant reduction of H2AX phosphorylation was observed between 26 and 30 h after cisplatin administration (Figure 1b).

We hypothesized that some kinases could negatively affect Wip1 activity. The inhibition of such kinases could increase Wip1 activity, simultaneously reducing *γ*H2AX phosphorylation and increasing sensitivity of p53-negative cells to this chemotherapeutic drug. Thus, we decided to perform a high-throughput screening for kinases, which may negatively regulate the activity of Wip1 upon DDR using the loss of phospho-Ser139 H2AX signal as a readout. To this end, we used the timepoint 28 h after CDDP treatment when the maximum activity of Wip1 towards Ser139 H2AX was detected in cisplatin-treated cells transfected with a siRNA kinome library. A 96-well format was used to monitor the phospho-Ser139 H2AX signal by flow cytometry system GUAVA.

To validate the system and set up the experimental conditions of high-throughput screening, we used siRNA to ATR kinase, which directly phosphorylates Ser139 on H2AX upon cisplatin-induced DNA damage.^28^ We showed that ATR inhibition significantly reduced the level of phospho-H2AX after cisplatin treatment (Supplementary Figure S2).

A library of siRNAs against 711 target kinases was used in this system. siRNAs that were able to change the number of *γ*H2AX-positive cells, and intensity of *γ*H2AX phosphorylation by at least 40% compared with control samples treated with non-targeting siRNA and same cisplatin concentration were arbitrarily set up as a threshold for positive hits (Figure 1c). Kinases, which are known as enzymes directly phosphorylating H2AX on Ser139, were excluded from further analysis.

**Figure 2:**
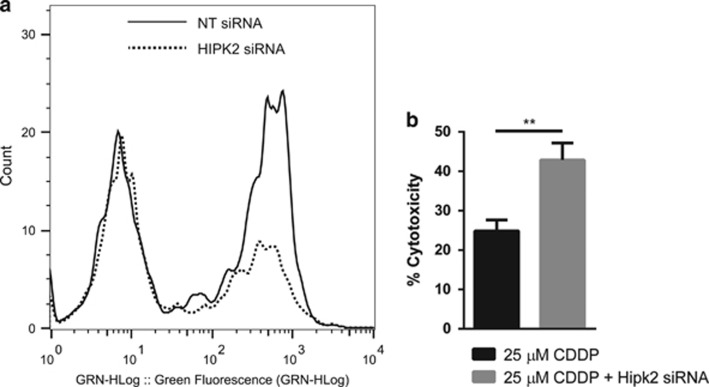
Hipk2 siRNA decreases H2AX phosphorylation but increases cisplatin toxicity in p53-negative Saos2 osteosarcoma cells. (**a**) Flow cytometry assay of H2AX phosphorylation of Saos2 osteosarcoma cells following a treatment with 25 *μ*M CDDP for 28 h after a reverse transfection of a non-targeting siRNA (line) or Hipk2 siRNA (dots). (**b**) Comparison of cytotoxicity following a 25 *μ*M treatment of CDDP for 72 h in Saos2 osteosarcoma cells with or without a transfection of Hipk2 siRNA

Interestingly, we noticed that the siRNA targeting Hipk2 was among siRNAs that reduced cisplatin-induced phosphorylation of H2AX and we confirmed this result (Figure 2a,Supplementary Figure S1C). Hipk2 kinase was recently reported as a negative regulator of Wip1.^29^ This confirms that our screen is able to identify regulators of Wip1, which also affect DNA damage-induced phosphorylation of H2AX (Supplementary Figure S3). Moreover, we observed a significant increase in Saos2 sensitivity towards cisplatin after depletion of Hipk2 kinase with specific siRNA (*P*=0.0037), which indicates that the ablation of Hipk2 restores Wip1 activity (Figure 2b).

### Wee1 inhibition decreases cisplatin-induced H2AX phosphorylation and sensitizes Saos2 cells to this drug

Among positive hits identified, we selected Wee1 kinase as a proof of principle that inhibition of a kinase could increase tumor cell sensitivity to chemotherapy without any increase in DNA damage signaling mark, that is, *γ*H2AX. Wee1 was never reported before as a kinase directly affecting *γ*H2AX phosphorylation.

**Figure 3:**
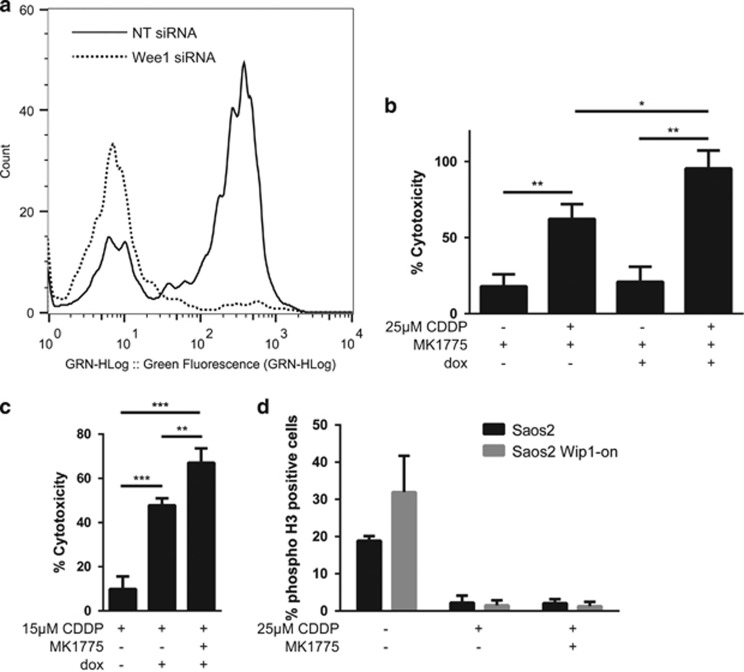
Wee1 partial inhibition potentializes Wip1-dependent cisplatin-induced cytotoxicity in p53-negative tumor cells but not by inducing a mitotic catastrophe. (**a**) Flow cytometry assay of H2AX phosphorylation of Saos2 osteosarcoma cells following a treatment with 25 *μ*M of CDDP for 28 h after a reverse transfection of a non-targeting siRNA (line) as a control, or Wee1 siRNA (dots). (**b**) Cytotoxicity assay in Saos2 osteosarcoma cells after inhibition of Wee1 by the chemical compound MK-1775 used at a low concentration of 75 nM 2 h prior a 25 *μ*M CDDP treatment for 72 h, in absence or presence of Wip1, induced by a 1 *μ*g/ml treatment of doxycycline 24 h prior to the chemotherapeutic treatment. (**c**) Cytotoxicity assay in Saos2 osteosarcoma cells after inhibition of Wee1 by 75nM MK-1775 2 h prior a 15 *μ*M CDDP treatment for 72 h, with or without Wip1, induced by a 1 *μ*g/ml treatment of doxycycline. (**d**) Immunofluorescence assay targeting the mitosis-specific marker phospho-histone H3. Cells were treated for 16 h with 100 ng/ml nocodazole to block them in mitosis, with triple combination of 25 *μ*M CDDP, 75nM Wee1 inhibitor MK-1775 and 1 *μ*g/ml doxycycline to induce Wip1 overexpression

We confirmed that siRNA to Wee1 decreased *γ*H2AX phosphorylation following cisplatin-induced DNA damage (Figure 3a, Supplementary Figures S1B and S3).

Recently, the chemical compound MK-1775 was identified as a specific inhibitor of Wee1 kinase activity. It was reported that this compound could sensitize several types of cancer cell lines to a number of chemotherapeutic agents.^30, 31, 32^ We demonstrated that MK-1775 potentiated Wip1-dependent sensitization to cisplatin in p53-negative Saos2 cells, even with a lower cisplatin concentration (*P*=0.0099). Indeed, by using triple combination (cisplatin+MK-1775+Wip1 overexpression), we were able to reduce the effective concentration of cisplatin by 40%, from 25 *μ*M effective concentration in cells with Wip1 overexpression alone to 15 *μ*M (Figures 3b and c). It is worth noting that we have used here a low non-toxic concentration of MK-1775, 75 nM, to avoid it acting as a single agent. At that concentration, it has been shown that MK-1775 can inhibit Wee1 only by 50%.^32^ On the contrary, in the absence of doxycycline-induced Wip1 overexpression, MK-1775-mediated sensitization (*P*=0.0201) of tumor cells to cisplatin was significantly attenuated.

Wee1 is a major positive regulator of G2 checkpoint. It has been suggested that Wee1 inhibition abolishes DNA damage-induced G2 arrest. During chemotherapy-induced DNA damage, Wee1 inhibition promotes premature entry of cells with unrepaired DNA into mitosis thereby inflicting cell death by mitotic catastrophe.^33^ Here, we demonstrated that the number of cells entering mitosis in population treated by the above-mentioned triple combination was similar to that observed in cells treated with cisplatin alone (Figure 3d). The preserved activity of pre-mitotic checkpoints can be explained by only partial inhibition of Wee1 in our settings or by activation of checkpoints earlier than G2 arrest. The p53-negative status of our model cell line, Saos2, indicates that G1 checkpoint should be compromised, because p53 is one of the major regulator of G1 arrest.^34, 35^

The cell cycle profile of cells treated with the different combinations (i.e., cisplatin alone, cisplatin plus MK-1775 or Wip1 overexpression, or triple combination) suggests that in triple and double drug combinations, most of the cells were entering S-phase DNA replication process. Moreover, we have shown that pro-apoptotic signaling is activated in S-phase, as shown in Supplementary Figure S4, by a co-localization of active caspase-3 with BrdU staining of S-phase population.

**Figure 4:**
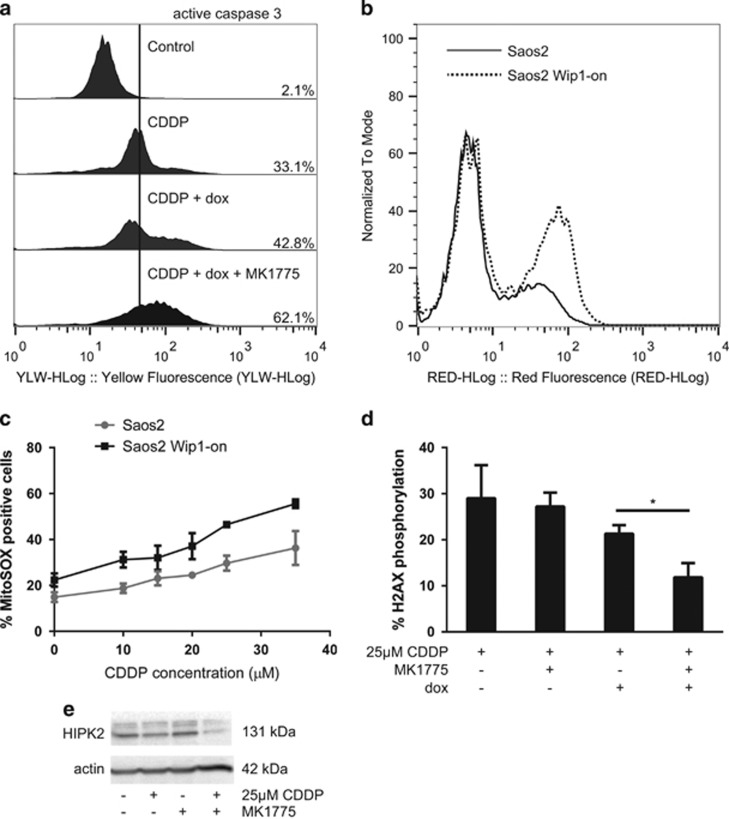
A caspase-3-dependent apoptosis correlated to mitochondrial superoxide production following cisplatin-induced DNA damage is Wip1-dependent in Saos2 osteosarcoma cells, and is related to Hipk2 deregulation by Wee1 kinase inhibition. (**a**) Anti-active caspase-3 flow cytometry assay following a treatment of Saos2 osteosarcoma cells with 25 *μ*M CDDP alone, with 1 *μ*g/ml doxycycline to induce Wip1 overexpression, or in triple combination with 75 nM MK-1775. (**b**) Flow cytometry profile showing the Wip1-dependency, induced by doxycycline, of mitochondrial ROS formation after a treatment with 25 *μ*M of CDDP for 24 h. (**c**) Flow cytometry assessment of mitochondrial superoxide production. Saos2 osteosarcoma cells were treated with increasing doses of CDDP for 24 h with or without induction of Wip1 by 1 *μ*g/ml doxycycline. Mitochondrial superoxide formation has been detected using MitoSOX superoxide indicator by flow cytometry after an incubation of cells with 2 *μ*M of the dye for 15 min at 37 °C. (**d**) Anti*-γ*H2AX flow cytometry assay following 28 h treatment of Saos2 osteosarcoma cells with 25 *μ*M CDDP, 75 nM MK-1775 and 1 *μ*g/ml doxycycline to induce Wip1 overexpression. (**e**) Western blot of anti-Hipk2 with or without a 25 *μ*M CDDP treatment and a Wee1 partial inhibition by 75 nM of MK-1775 for 20 h

Figure 4a shows that cells treated according to our scheme, CDDP, MK-1775 and Wip1 overexpression, die by a caspase-3-dependent apoptosis, highlighting the efficiency of the triple combination. The efficacy of this new treatment was also confirmed with an increase in cleaved PARP levels after the treatment (Supplementary Figure S5). The oxidation of mitochondria, detected with MitoSOX red fluorescent reagent, was increased also in a cisplatin- and Wip1-dependent manner (Figures 4b and c). Hence, cell death in Saos2 cells upon Wip1 overexpression involves the mitochondrial pathway and has strong correlation with mitochondrial oxidation. Further, we have found that Wee1 inhibitor MK-1175 reduced cisplatin-induced *γ*H2AX phosphorylation in a Wip1-dependent manner (Figure 4d, *P*=0.0103), because MK-1775 has no impact on H2AX phosphorylation by itself in cells without Wip1 overexpression. This indicates that Wee1 could negatively affect Wip1 activity. Inhibition of Wee1 by MK-1775 can activate Wip1 and increase p53-negative cell sensitivity towards cisplatin. Interestingly, simultaneous treatment with CDDP and MK-1775 destabilized Hipk2 kinase, the negative regulator of Wip1 activity, confirming that Wee1 is involved in the negative regulation of Wip1 pathway (Figure 4e).

### Inhibition of Wee1 combined with Wip1 overexpression does not sensitize normal cells to cisplatin

**Figure 5:**
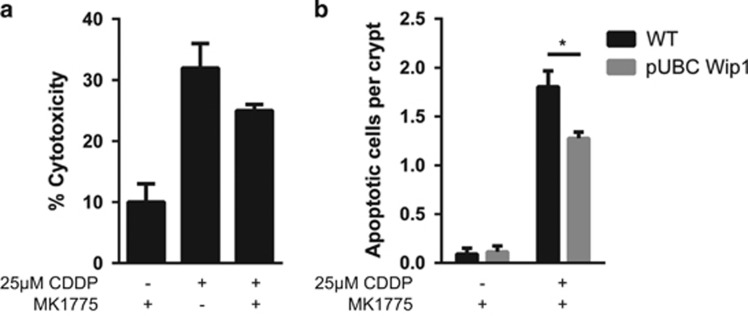
Wee1 inhibitor MK-1775 does not increase cell death by itself *in vitro* or *in vivo* without combination with cisplatin. (**a**) Cytotoxicity assay in MEF cells following 75 nM MK-1775 and 25 *μ*M CDDP treatment for 72 h. (**b**) Immunohistochemistry of intestine slides of wild-type mice (WT) and constitutively Wip1-expressing transgenic mice (pUBC-Wip1) after being treated *per os* with 30 mg/kg MK-1775 2 h prior an intraperitoneal injection of 10 mg/kg CDDP. Apoptotic cells were stained with an anti-cleaved caspase-3 monoclonal antibody, and were counted in at least 100 intestine crypts per mice

Conventional chemotherapy can severely affect several normal tissues and organs that could lead to severe side effects during and after anti-cancer treatment.^36, 37, 38^ We found that our proposed triple combination did not significantly increase cell death of normal mouse embryonic fibroblasts (Figure 5a). Moreover, treatment of wild-type and Wip1 transgenic mice showed that MK-1775 used at a concentration proposed for anti-cancer treatment (30 mg/kg) is not toxic for fast proliferating tissues such as intestinal epithelium. Furthermore, MK-1775, when added with cisplatin, does not disrupt Wip1-protective effect of normal tissues during chemotherapy,^6^ as less apoptotic cells were observed in pUBC-Wip1 mice compared with WT mice after MK-1775 and 10 mg/kg CDDP treatment (Figure 5b, Supplementary Figure S6).

### Wip 1 positively affects survival of patients with p53-negative colorectal tumors

**Figure 6:**
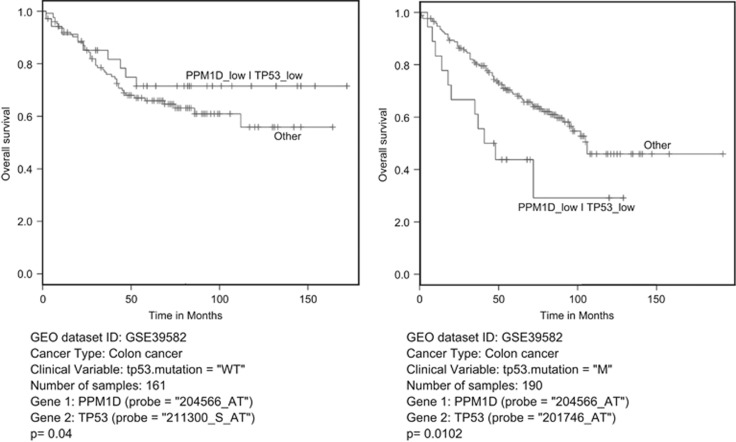
Wip1 positively affects survival of patients with p53-negative colorectal tumors. Bioinformatics analysis of two cohorts of patients with colon cancer. Correlation effects between overall survival of patients and PPM1D gene expression with p53 status was studied from an Affymetrix gene expression dataset, using standard A-package.

To assess the biological significance of PPM1D gene expression in tumors with variable p53 status, we used a bioinformatics approach on clinical gene expression data of colorectal cancer patients. Among the patients, 190 samples corresponded to patients with a mutant status of p53 and 161 samples contained wild-type p53. These two cohorts were examined separately for the correlation between PPM1D expression and survival of patients. In the presence of wild-type p53, low level of PPM1D expression correlated with better prognosis, which is consistent with the oncogenic role of PPM1D (Figure 6). In contrast, in the presence of mutant p53, low level of PPM1D expression was associated with poor survival of colon cancer patients, especially after 75 months. This result supports, although indirectly, the positive role of PPM1D in the absence of functional p53. Collectively, these results suggest that Wip1 has opposite roles in tumors with wild-type p53 versus the ones with mutant p53.

## Discussion

Platinum derivatives, including cisplatin, are widely used chemical agents in the treatment of solid malignancies like neck, lung, testis, ovarian or testis tumors. Their efficacy is dose-dependent, but also are the side effects, particularly nephrotoxicity. Indeed, the main complication after cisplatin chemotherapy is acute kidney injury. This is an important obstacle to the use of doses that maximize its antineoplastic properties.^39^ Moreover, when the p53 tumor suppressor is mutated and inactive, which is the case in more than half of cancers, the effectiveness of such treatments is significantly compromised.^41^ This is why restoring tumor cell sensitivity to anti-cancer agents in p53-negative tumors and at the same time protecting normal tissues from chemotherapy-induced side effects, or at least decreasing toxicity towards them, is a very attractive strategy in oncology. The Wip1 activation in p53-negative cells was a first example of such kind of strategy. Indeed, our team has shown that Wip1 overexpression in p53-negative tumor cells fulfills these two objectives, by both promoting caspase-dependent apoptosis in tumors and inhibiting p53-regulated apoptosis in healthy normal tissues.^6, 10^

In this study, as a strategy to potentiate the effect of Wip1 overexpression in p53-negative cancers, we have designed a high-throughput screening of the human kinome and analyzed p53-negative cells for kinases, whose inhibition could decrease cisplatin-induced H2AX phosphorylation and at the same time increase tumor cell sensitivity to chemotherapy. H2AX was reported in several publications as a target of Wip1 enzymatic activity.^16, 17, 18, 19, 20^ One of our aims was to identify kinases that negatively regulate Wip1 pathway, so their depletion or inhibition could lead to an increased Wip1 activity and, consequently, to a reduced DNA damage signaling through H2AX and to an elevated sensitivity of p53-negative cells to chemotherapeutic drugs. We confirmed data published by Choi *et al.*,^29^ demonstrating that Hipk2 kinase can affect H2AX phosphorylation in a Wip1-dependent manner. In p53-negative cells, we observed an increase in sensitivity to chemotherapeutical agent after depletion of Hipk2 kinase, mimicking the phenotype observed by Wip1 overexpression in the same p53-negative type of cells.

As a result of our screen, we identified Wee1 tyrosine kinase, which was previously described as an effector of G2 checkpoint regulation by phosphorylating Cdc2,^43^ but not as a kinase directly affecting H2AX phosphorylation. siRNA-mediated depletion of Wee1 or treatment with the specific Wee1 inhibitor MK-1775 not only reduced cisplatin-induced H2AX phosphorylation in p53-negative Saos2 osteosarcoma cells, but also significantly potentiated Wip1-dependent sensitization to this chemotherapeutic drug. Importantly, we were able to achieve a reduction of the effective concentration for both drugs, CDDP and MK-1775, used in triple combination with Wip1 overexpression without diminishing their anti-tumor toxicity. This result is very interesting from the perspective of minimization of cisplatin side effects, especially acute kidney injury. Furthermore, it enhances the Wip1 overexpression protective effect on the normal tissues.

The very low non-toxic concentration of MK-1775 we used can inhibit only about 50% of Wee1 enzymatic activity.^32^ At this concentration, G2 checkpoint was preserved and cells accumulate in S-phase after CDDP treatment as it was shown for Wip1-dependent sensitization of Saos2 cells.^6^ Alternatively, our data indicate that MK-1775 at low concentration could activate Wip1 pathway through deregulation of Hipk2 activity.

The effect of Wip1 overexpression depends on the p53 status of the tumor. Our *in vitro* data on the positive effects of combinatorial treatment with overexpression of Wip1, Wee1 inhibition and chemotherapy are reinforced by the bioinformatics analyses of patients with colorectal cancer. Of note, it has been shown that wild-type p53 inhibited Wee1 expression through miR-26a.^44^ In tumors, where PPM1D is overexpressed, p53-mediated expression of miR-26a is attenuated. Accordingly, we found that high expression of PPM1D in wild-type p53 tumors correlates with poor prognosis, which is in a good agreement with previously published data.^45, 46^ This justifies the development of Wip1 inhibitors to enhance p53-positive tumors treatments, such as neuroblastoma.^11^ In contrast, in p53-negative tumors, high expression of PPM1D correlates with better prognosis. This result is in line with our approach of overexpression of PPM1D in p53-negative tumors to restore chemotherapy efficiency and at the same time protecting normal tissues from side effects of the treatment. This is why developing activators of PPM1D gene expression to overexpress Wip1 is as important as its inhibitor counterpart. We believe that this new combination of Wip1 overexpression with MK-1775 chemical inhibitor, which is already in clinical trial, represents a novel and promising approach in treating patients harboring p53-negative tumors.

## Material and Methods

### Cell lines and cell culture

The Saos2 Tet-on cell line, human osteosarcoma cells with a tetracyclin-inducible gene expression system were purchased from Clontech, Mountain View, CA, USA. The Saos2 cells were modified to stably express Wip1 following a treatment with doxycycline (Sigma–Aldrich, St. Louis, MO, USA, D9891), as described in a previous paper.^6^ Murine embryonic fibroblasts were isolated according to the standard protocol from wild-type 12-day c57-Bl/6 mice. All cell lines were cultured at 37 °C with 5% CO_2_ in a humidified incubator in DMEM high glucose (Dutscher, Brumath, France, L0104-500) with 10% FBS (Pan Biotech, Aidenbach, Germany, 8500-P131704) and Penicillin-Streptomycin-Amphotericin antibiotics (Pan Biotech, P06-07300). To induce Wip1, cells were treated with 1 *μ*g/ml doxycycline 24 h prior to cisplatin (Sigma–Aldrich, P4394) and/or 75nM MK-1775 Wee1 inhibitor treatments (Axon Medchem, Groningen, The Netherlands, 955365-80-7).

### High-throughput screening of human kinome and anti-*γ*H2AX assay

Cells were reverse-transfected in 96-well plates with a Dharmacon siGENOME SMARTpool siRNA library of human protein kinases (Dharmacon, GE Healthcare Europe GmbH, Vélizy-Villacoublay, France, G-003505), a fluorescent oligonucleotide or a non-targeting siRNA as a transfection control. Two days after, the transfection condition was monitored with a fluorescent oligonucleotide duplex (Dharmacon, GE Healthcare Europe GmbH, D-001630-02-05), and cells were treated with 25 *μ*M cisplatin. Twenty-eight hours after cisplatin treatment, cells were fixed with 4% PFA (Sigma–Aldrich, P6148-500) for 20 min, then permeabilized with 90% methanol on ice for 30 min before blocking and staining with a Alexa488-conjugated anti-H2AX monoclonal antibody (BD Pharmingen, San Diego, CA, USA, 560445). After immunostaining, cells were resuspended in PBS for the high-throughput flow cytometry assay performed with the Guava EasyCyte flow cytometer (Merck Millipore, Darmstadt, Germany). Data obtained were analyzed using the FlowJo software. Wells transfected with a siRNA showing a modification of at least ±40% of H2AX phosphorylation compared with non-targeting siRNA-transfected samples were classified as positive hits.

### Cytotoxicity assay

Cytotoxicity of treatments have been assessed with a modified LDH-based production protocol using Promega Cytotox 96 Non-radioactive kit (Promega, Madison, WI, USA, G1780), following the manufacturer's protocol. Cytotoxicity assessment protocol has been modified following the instructions of Smith *et al.*^48^

### Phospho-histone H3 immunofluorescence

Cells were treated with 75 nM MK-1775 2 h before adding 100 ng/ml Nocodazole (Selleck Chemicals, Houston, TX, USA, S2775) and 25 *μ*M cisplatin for 16 h. Cells were then fixed in 4% PFA for 20 min and blocked in 1 × PBS containing 3% BSA and 0.3% Triton X-100 (Sigma–Aldrich, T8787-250) for 1 h. Alexa488-conjugated anti-phospho-H3 monoclonal antibody (Cell Signaling, Danvers, MA, USA, 9708S) has been used at a 1:50 dilution and samples were incubated overnight at 4 °C. Coverslips have been rinsed three times with 1 × PBS, then once in deionized water and mounted on slides with a DAPI-containing mounting medium (Vector, Burlingame, CA, USA, H-1200).

### MitoSOX mitochondrial superoxide production assay

Mitochondrial superoxide formation was detected by flow cytometry using the MitoSOX fluorogenic dye (Thermo Fisher Scientific, Waltham, MA, USA, M36008). Briefly, after chemotherapeutic treatment of cells with CDDP, from 0 to 35 *μ*M for 24 h, cells were collected and resuspended in HBSS (Dutscher, L0611-500) containing 2 *μ*M MitoSOX reagent. After 15 min of incubation at 37 °C in the dark, cells were washed two times in PBS before analysis on the Guava EasyCyte cytometer (Merck Millipore, Darmstadt, Germany).

### *In vivo* cytotoxicity assay of combinatory treatment

Wild-type and transgenic mice, described previously,^49^ had access to food and water *ad libitum*. Animal experiments were performed following guidelines of the Federation of European Laboratory Animal Science Associations. Mice received MK-1775 inhibitor at a dose of 30 mg/kg *per os* diluted in 0.5% methylcellulose (Sigma–Aldrich, M0262). Three hours after, 10 mg/kg CDDP were injected intraperitoneally. Twelve hours later, mice were killed and intestines were collected, fixed in formol for 24 h before being stored in 70% ethanol until immunohistochemistry experiment, performed with an anti-caspase-3 monoclonal antibody (R&D systems, Minneapolis, MN, USA, AF835) and visualized using Dako EnVision System-HRP (Dako, Carpinteria, CA, USA, K4010).

### Clinical data correlation analysis of p53 status and PPM1D gene expression with survival of patients

A bioinformatics approach has been used, by using an Affymetrix gene expression dataset from 566 patients with colon cancer. Two cohorts have been studied, depending on the p53 status of the patients, wild-type or mutated. Correlation effects of PPM1D expression together with p53 status and survival of patients was calculated using standard A-package.

### Statistics

All data are expressed as the mean±S.E.M. and experiments have been independently repeated at least three times. Differences between two groups were assessed by Student's unpaired *t*-test performed with GraphPad Prism 6 software and data were considered significant if *P*<0.05.

## Abbreviations

ATM: Ataxia telangiectasia mutated
ATR: Ataxia telangiectasia and Rad3-related protein
BrdU: Bromodeoxyuridine
Cdc2: Cell division control protein 2 homolog
CDDP: cis-diaminedichloroplatine(II)
DDR: DNA damage response
DNA: Deoxyribonucleic acid
DNA-PK: DNA-dependent protein kinase
H2AX: histone H2A family member X
Hipk2: Homeodomain-interacting protein kinase 2
PARP: Poly ADP-ribose polymerase
PPM1D: Protein phosphatase Mg^2+^/Mn^2+^ dependent 1D
RNA: Ribonucleic acid
Saos: Sarcoma osteogenic
Ser: Serine
siRNA: Small interfering RNA
Wee1: Wee1 G2 checkpoint kinase
Wip1: Wild-type p53-induced phosphatase 1
