# Supplementary material for: Wee1 inhibition potentiates Wip1-dependent p53-negative tumor cell death during chemotherapy
Source: Cell Death Dis. 2016 Apr 14;7(4):e2195–. doi: 10.1038/cddis.2016.96 (PMC4855675; doi:10.1038/cddis.2016.96)
Supplement: Supplementary Information [file cddis201696x1.docx]

SUPPLEMENTARY MATERIAL

**γH2AX immunofluorescence**

Cells were treated either with 1 µg/mL doxycycline for 24 hours, or transfected with Wee1, Hipk2 SMARTpool siRNA or NT siRNA (Dharmacon, GE Healthcare Europe GmbH, France, M-005050-02, M-003266-03 and D-001210-02-05 respectively). 48 hours after transfection, the cells were treated with 15 µM CDDP for 28 hours before performing immunofluorescence against γH2AX. Cells were fixed in 4% PFA for 20 minutes and blocked in 1X PBS containing 3% BSA and 0.3% Triton X-100 (Sigma-Aldrich, USA, T8787-250) for 1 hour. Alexa488-conjugated anti-H2AX monoclonal antibody (BD Pharmingen, USA, 560445) has been used at a 1:200 dilution and samples were incubated overnight at 4°C. Coverslips have been rinsed three times with 1X PBS, then once in deionized water and mounted on slides with a Dapi-containing mounting medium (Vector, H-1200).

**BrdU and active caspase-3 flow cytometry**

Flow cytometry analysis of BrdU/active caspase-3 was done in Saos2 osteosarcoma cells. The triple combination (15 µM CDDP, 1 µg/mL doxycycline and 75 nM MK-1775) was applied to cells for 48 hours. The BD-Pharmingen BrdU flow kit (BD Pharmingen, USA, 552598) was used, with APC anti-BrdU and PE anti active-caspase 3 antibodies (BD Pharmingen, USA, 51-23619L and 550821 respectively). The experiment was performed following manufacturer’s protocol.

**Western Blot antibodies**

Wip1 monoclonal antibody was puchased from Santa Cruz (Santa Cruz, USA, sc376257). Wee1 antibody was purchased from Cell Signaling (CST, USA, 13084T). Hipk2 antibody has been provided by Dr. Ettore Appella (NIH, Bethesda, USA). Cleaved PARP was purchased from Cell Signaling (CST, USA, 5625).

SUPPLEMENTARY FIGURES LEGENDS

**Figure S1: Protein levels of Wip1, Wee1 and Hipk2 after overexpression or depletion with siRNA.** (A) Western blot showing level of Wip1 in the Saos2 Wip1-ON cell line vs Saos2 after a treatment with 1 µg/mL doxycycline for 24 hours. (B, C) Western blot showing level of Wee1 and Hipk2 after their depletion induced by a SMARTpool siRNA reverse-transfection. 48 hours after transfection, cells were collected, lysed and protein extract was compared to samples transfected with non-targeting control siRNA.

**Figure S2: ATR inhibition decreases cisplatin-induced H2AX phosphorylation.** H2AX phosphorylation is decreased by ATR inhibition induced by a reverse transfection of Saos2 osteosarcoma cells with a SMARTpool ATR siRNA. 48 hours after transfection, cells have been treated with 25 µM CDDP for 28 hours, then H2AX phosphorylation have been measured by flow cytometry.

**Figure S3: Wip1 overexpression or Wee1 and Hipk2 inhibition decrease cisplatin-induced H2AX phosphorylation.** Representative immunofluorescence images of Saos2 cells showing the impact of Wip1, Wee1 and Hipk2 on H2AX phosphorylation following DNA damage. Wip1 was induced by a treatment with 1 µg/mL doxycycline for 24 hours. Wee1 and Hipk2 were both inhibited by a SMARTpool siRNA reverse-transfection. 48 hours after transfection, the cells were treated with 15 µM CDDP for 28 hours before performing immunofluorescence against γH2AX, and compared to cells transfected with non-targeting control siRNA.

**Figure S4: Cisplatin and MK-1775 treatment triggers pro-apoptotic signaling in Saos2 Wip1-ON cells resulting in caspase-3 activation in S-phase.** Flow cytometry analysis of BrdU/active caspase-3 co-staining of Saos2 cells. The triple combination (15 µM CDDP, 1 µg/mL doxycycline and 75 nM MK-1775) was applied to cells for 48 hours. 1 hour before the end of the treatment, 10 µM of BrdU were added to the culture medium.

**Figure S5: Triple combination is efficient in inducing cleaved-PARP in p53-negative Saos2 cells.** Western blot showing level of cleaved-PARP in Saos2 osteosarcoma cells after 15 µM CDDP ± 1 µg/mL doxycycline ± 75 nM MK-1775 for 48 hours, compared to non treated control cells. Densitometry analysis has been done with ImageJ software.

**Figure S6: Wee1 inhibitor MK-1775 does not affect the protective effect of Wip1 overexpression in mice intestines.** Representative images of immunohistochemical analysis of active caspase-3 staining in intestinal crypts of wild type mice (WT) and constitutively Wip1-expressing transgenic mice (pUBC-Wip1) after being treated *per os* with 30 mg/kg MK-1775 2 hours prior an intraperitoneal injection of 10 mg/kg CDDP.
