# Supplementary figures and images for: Wee1 inhibition potentiates Wip1-dependent p53-negative tumor cell death during chemotherapy
Source: Cell Death Dis. 2016 Apr 14;7(4):e2195–. doi: 10.1038/cddis.2016.96 (PMC4855675; doi:10.1038/cddis.2016.96)

A

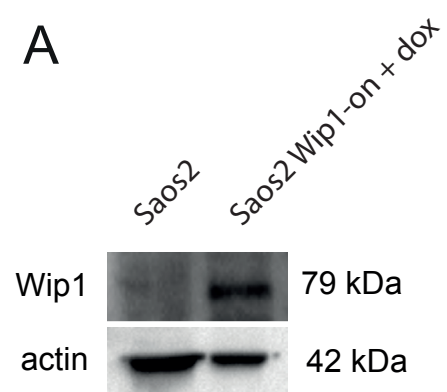

B

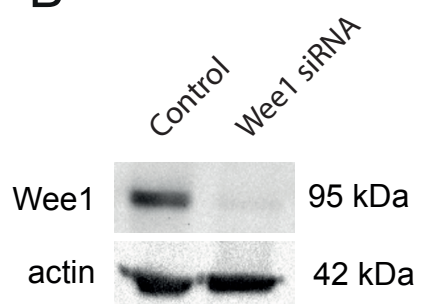

C

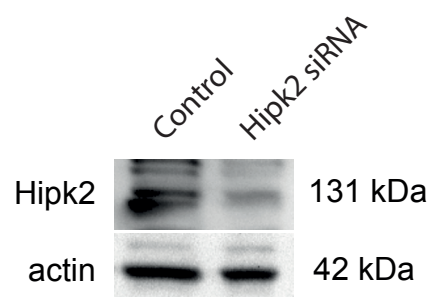

Supplement: Supplementary Figure 1 [file cddis201696x2.pdf]

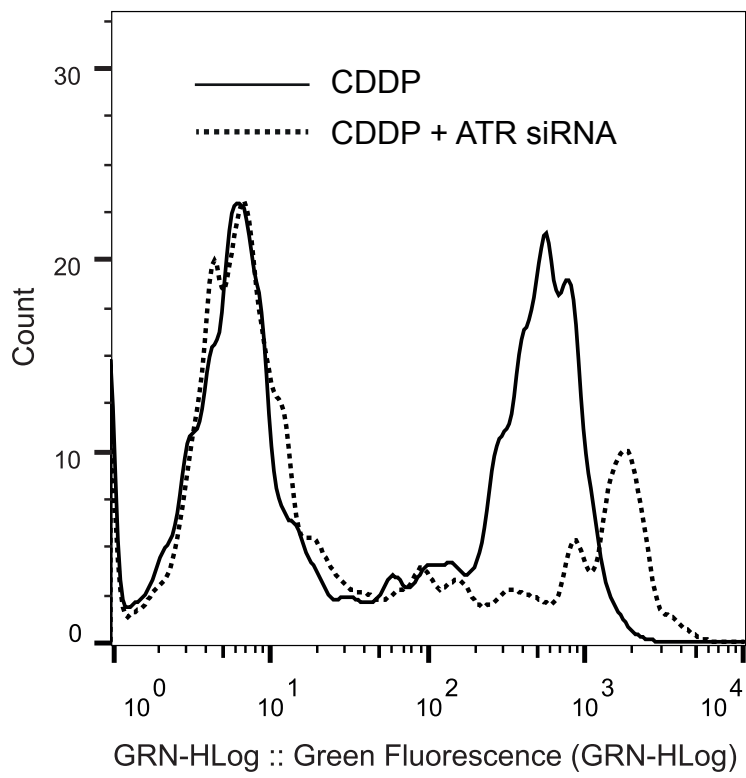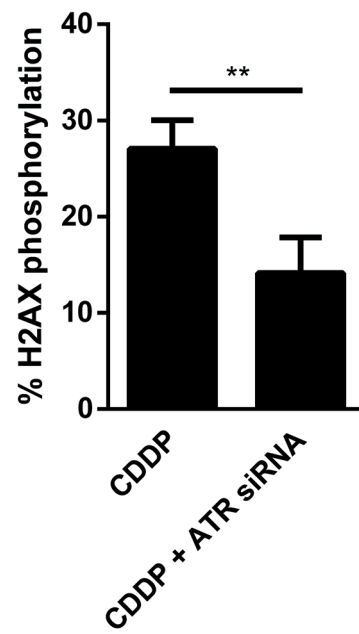

Supplement: Supplementary Figure 2 [file cddis201696x3.pdf]

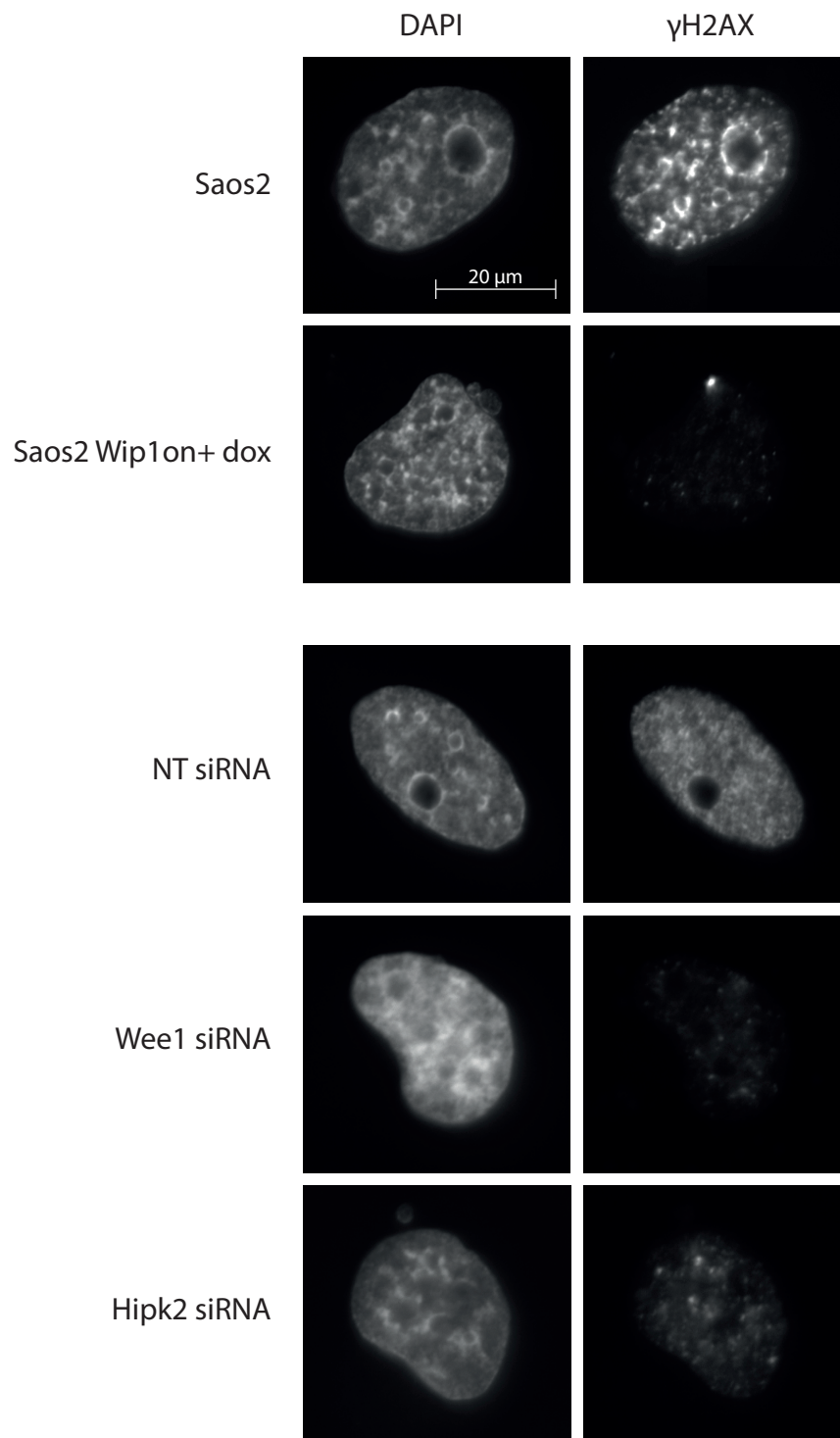

Supplement: Supplementary Figure 3 [file cddis201696x4.pdf]

active caspase 3

Control

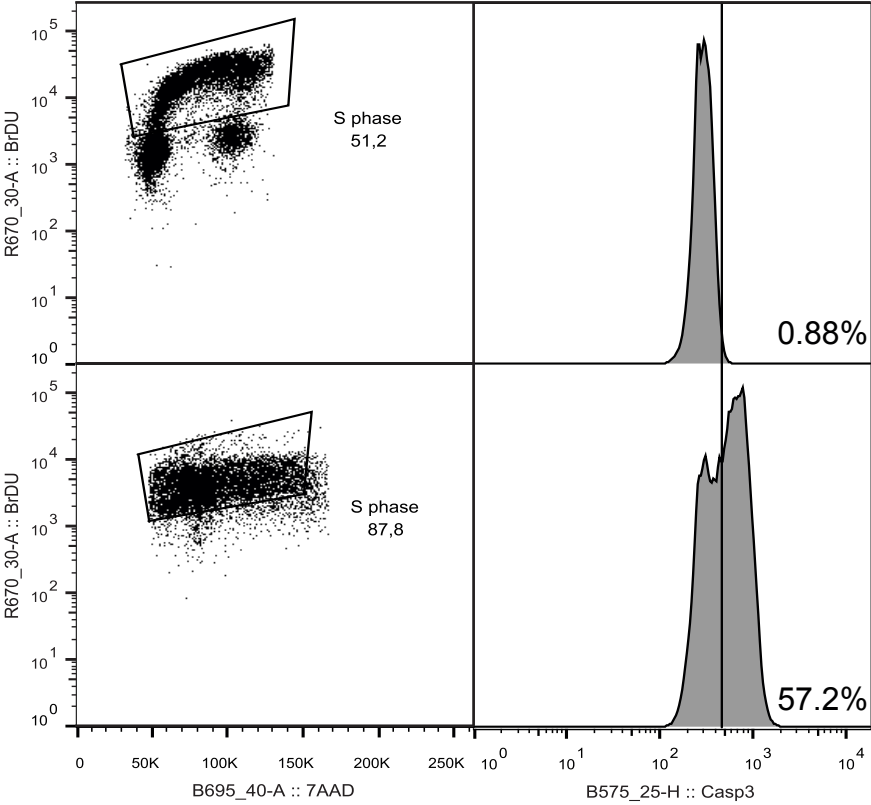

CDDP  
+ dox  
+ MK1775

Supplement: Supplementary Figure 4 [file cddis201696x5.pdf]

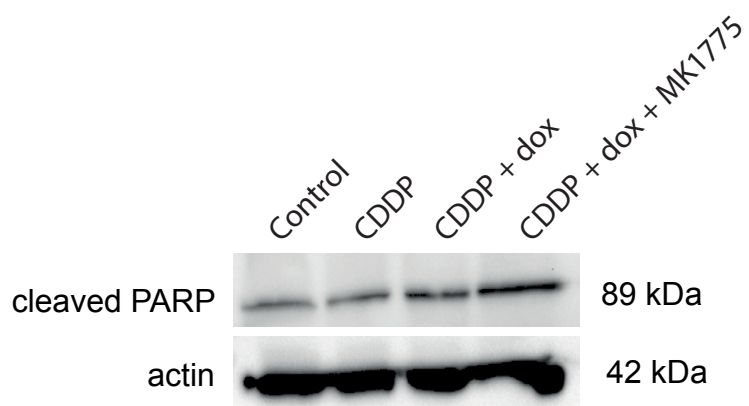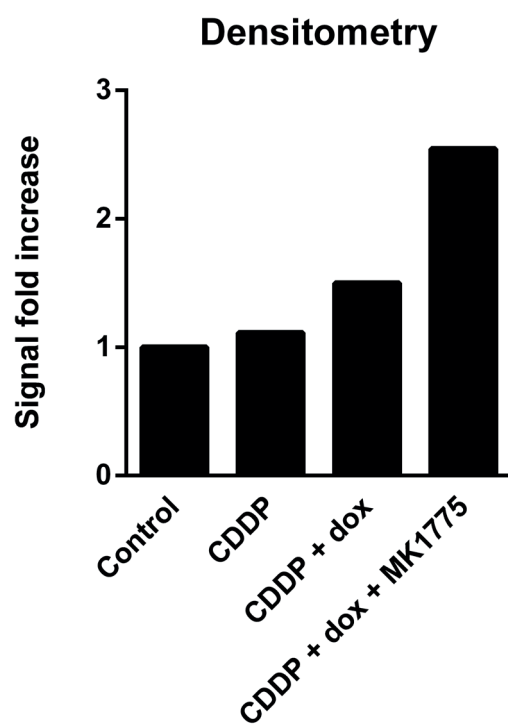

Supplement: Supplementary Figure 5 [file cddis201696x6.pdf]

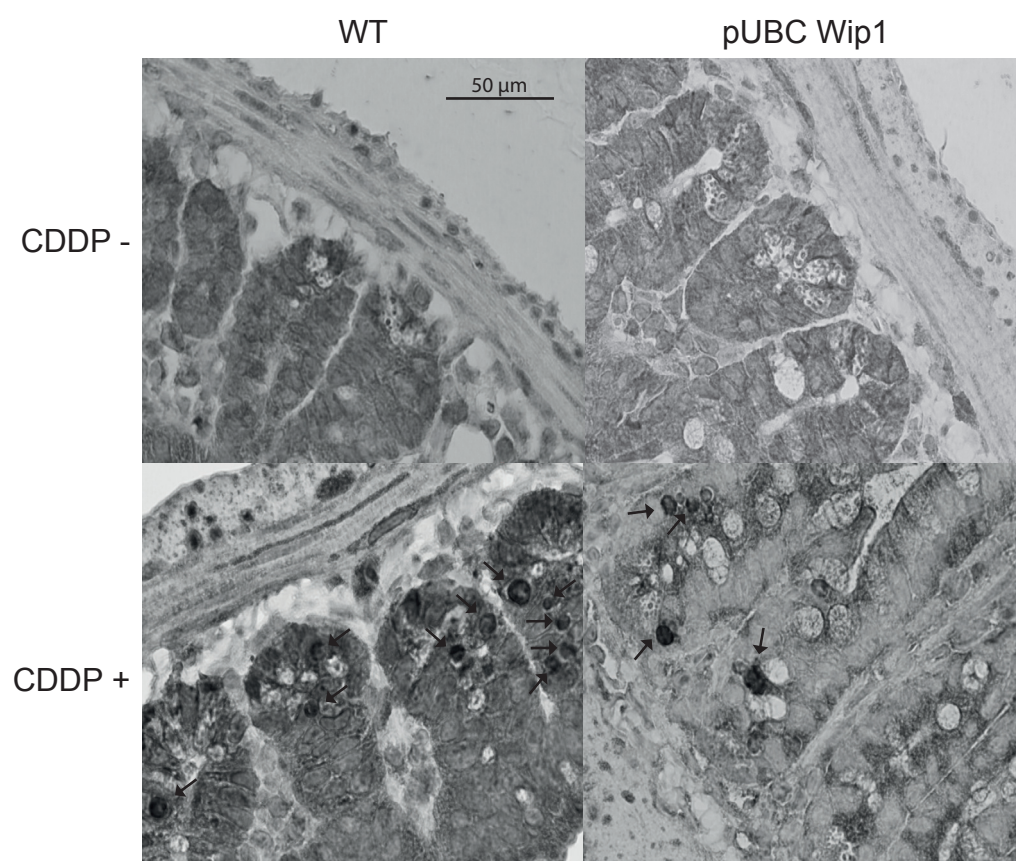

Supplement: Supplementary Figure 6 [file cddis201696x7.pdf]
